# Confocal Laser Scanning Microscopy for Analysis of *Pseudomonas aeruginosa* Biofilm Architecture and Matrix Localization

**DOI:** 10.3389/fmicb.2019.00677

**Published:** 2019-04-02

**Authors:** Courtney Reichhardt, Matthew R. Parsek

**Affiliations:** Department of Microbiology, University of Washington, Seattle, WA, United States

**Keywords:** biofilm, *Pseudomonas aeruginosa*, confocal laser scanning microscopy, exopolysaccharides, flow-cell

## Abstract

Most microbes can produce surface-associated or suspended aggregates called biofilms, which are encased within a biopolymer-rich matrix. The biofilm matrix provides structural integrity to the aggregates and shields the resident cells against environmental stressors, including antibiotic treatment. Microscopy permits examination of biofilm structure in relation to the spatial localization of important biofilm matrix components. This review highlights microscopic approaches to investigate bacterial biofilm assembly, matrix composition, and localization using *Pseudomonas aeruginosa* as a model organism. Initial microscopic investigations provided information about the role key matrix components play in elaborating biofilm aggregate structures. Additionally, staining of matrix components using specific labels revealed distinct positioning of matrix components within the aggregates relative to the resident cells. In some cases, it was found that individual matrix components co-localize within aggregates. The methodologies for studying the biofilm matrix are continuing to develop as our studies reveal novel aspects of its composition and function. We additionally describe some outstanding questions and how microscopy might be used to identify the functional aspects of biofilm matrix components.

## Introduction

Microbes form multicellular communities called biofilms (Costerton et al., 1995). Within these communities, microbial aggregates are encased in a biopolymer-rich extracellular matrix. Biofilm formation helps microbes to persist in several niches ranging from the natural environment to human infections (Costerton et al., 1999; Hall-Stoodley et al., 2004; Flemming et al., 2016). In the human host, biofilms cause serious and chronic infectious diseases including recurrent urinary tract infections, biofouling of medical devices, and chronic infections of wounds and burns (Donlan and Costerton, 2002; Parsek and Singh, 2003; Metcalf and Bowler, 2013). Typically, biofilm microbes exhibit decreased susceptibility to antimicrobial treatments relative to their planktonic counterparts (Stewart and Costerton, 2001). Biofilm matrix composition varies depending upon the microbial species and growth conditions. In general, biofilm matrix contains exopolysaccharides (EPS), proteins, and extracellular DNA (eDNA). These matrix components are assembled into supramolecular structures that aid in shielding microbes from external stresses (Flemming and Wingender, 2010). The matrix fills more than just a structural role. For example, the matrix can retain protective proteins (e.g., ecotin) or serve as a signal (e.g., to increase biofilm matrix production) (Irie et al., 2012; Steinberg and Kolodkin-Gal, 2015; Dragoš and Kovács, 2017; Tseng et al., 2018).

Investigations of biofilm matrix composition and structure are challenging due to their inherent complexity. Despite this challenge, several key microscopy and biochemical assays have been developed and successfully applied to annotate biofilm matrix composition and determine the functional roles of the matrix components (Azeredo et al., 2017). Genetic approaches have identified genes that are important for biofilm matrix formation (O’Toole et al., 1999). Putative biofilm-involved genes can be deleted and/or overexpressed, and then the resulting matrix composition and biofilm phenotypes can be assayed. For example, quantitative composition can be profiled using mass spectrometry methods (e.g., glycomics, proteomics, etc.) (Sauer, 2003; Zarnowski et al., 2014) or nuclear magnetic resonance (NMR) (Reichhardt and Cegelski, 2014). Changes in levels of known matrix components can be determined using immunoblotting (Fong et al., 2010; Colvin et al., 2012). The ability of bacteria to form communities can be measured in several ways including monitoring the ability of bacteria to aggregate in liquid culture (Borlee et al., 2010; Rybtke et al., 2015; Cooley et al., 2016; Reichhardt et al., 2018), form pellicles (biofilms at the air liquid interface) (Hollenbeck et al., 2014, 2016), and adhere to abiotic and biotic surfaces (Colvin et al., 2012). All these approaches involve generating average values for the entire community, while providing no information regarding heterogeneity in the system.

**Figure 1:**
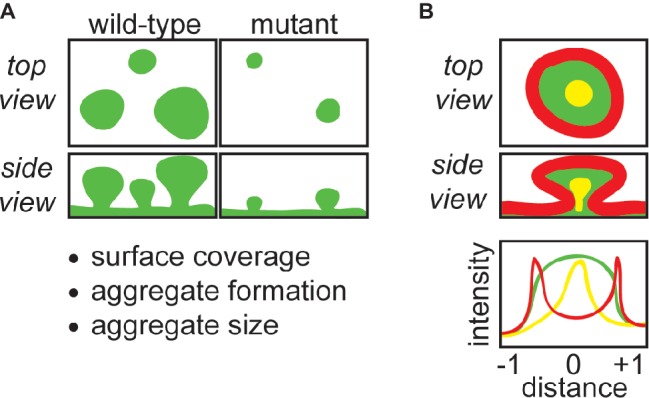
CLSM can be used to obtain important structural and functional information about biofilm matrix. **(A)** CLSM can be used to compare wild-type with matrix mutant strains grown in flow-cells, which provides information about how specific matrix components contribute to the amount of biomass covering the surface of the flow-cell coverslip, biofilm aggregate formation, and the morphology and size of aggregates. **(B)** The retention and localization of matrix components (e.g., EPS, eDNA) or exogenous molecules (e.g., antibiotics) can be monitored by CLSM. For example, EPS localization can be tracked with fluorescently-conjugated lectins, and the retention of antibiotics can be monitored by using fluorescently-conjugated antibiotics. Fluorescence intensity can be quantitated using image processing software, and correlated to position across the diameter of a biofilm aggregate. In the schematic, red-stained matrix elements localize to the periphery of the aggregate, green-stained elements are present throughout, and yellow-stained matrix elements localize to the aggregate interior.

Confocal laser scanning microscopy (CLSM) is a useful tool to study biofilms, and our lab has extensively applied CLSM to study biofilms cultured under continuous flow in flow-cell reactors (Borlee et al., 2010; Colvin et al., 2012; Tseng et al., 2013; Jennings et al., 2015). This method has several advantages including that it is a reproducible biofilm culturing format, live biofilms can be non-destructively imaged at multiple timepoints, and spatial information can be obtained regarding cell and matrix distribution (Heydorn et al., 2000a). Additionally, antibody- and lectin-conjugated dyes can be used to identify and spatially resolve biofilm constituents. CLSM in conjunction with image analysis software such as COMSTAT (Heydorn et al., 2000b) can be used to quantitatively study biofilm matrix, as well as the amount of adherent biomass, while localization and relative amounts of matrix constituents can also be determined (Swerhone et al., 2001; Berk et al., 2012). Additionally, spatiotemporal effects of different nutrient environments or antimicrobial treatments can be monitored (Tseng et al., 2013; Sønderholm et al., 2017). This review will discuss ways that our laboratory has implemented CLSM to study matrix composition and function of *Pseudomonas aeruginosa* biofilms, with our general approach summarized in Figure 1.

*P. aeruginosa* is both a model organism for laboratory study of biofilms and an important pathogen that causes chronic infections. Examples of chronic infections caused by *P. aeruginosa* include respirator-associated pneumonia, infections of burns and wounds, and lung infections in patients with cystic fibrosis (CF) (Mulcahy et al., 2014). *P. aeruginosa* can use three EPS to assemble its biofilms: alginate, Psl, and Pel. Alginate is the key EPS of mucoid biofilms, and Psl and Pel are predominant in non-mucoid biofilms (Mann and Wozniak, 2012; Moradali et al., 2017). Alginate is a negatively charged polymer of guluronic and mannuronic acids (Schürks et al., 2002). Psl is a neutral polysaccharide consisting of pentasaccharide repeats containing D-mannose, D-glucose, and L-rhamnose (Kocharova et al., 1988; Ma et al., 2007; Byrd et al., 2009). Pel is a cationic polymer of partially deacetylated N-acetylglucosamine and N-acetylgalactosamine (Jennings et al., 2015). These EPS are present in varying amounts depending upon the specific strain and stage of infection (Ma et al., 2006; Colvin et al., 2012). For example, alginate, Psl, and Pel are believed to be expressed during different stages of *P. aeruginosa* CF lung infections (Martin et al., 1993).

Proteins also play a key role in the *P. aeruginosa* biofilm matrix. The structural matrix protein CdrA is important for aggregate assembly and localization and retention of Psl (discussed in detail below) (Borlee et al., 2010; Reichhardt et al., 2018). Additional proteins have been found in the matrix, which could impart functions ranging from nutrient acquisition to protection from oxidative stress (Toyofuku et al., 2012). Recently, the serine-protease inhibitor ecotin was identified as a matrix protein that binds to Psl (Tseng et al., 2018). Matrix-bound ecotin was found to protect bacteria from attack by the host immune protease neutrophil elastase. In these ways, the biofilm matrix acts as both a structural scaffold for biofilm assembly and an active functional network.

Finally, eDNA has been identified as a component of biofilms formed by several species, including *P. aeruginosa* (Whitchurch et al., 2002; Moscoso et al., 2006; Rice et al., 2007; Harmsen et al., 2010). The source of eDNA in *P. aeruginosa* is unclear although it may simply result from cell lysis that occurs during biofilm growth (Webb et al., 2003; Allesen-Holm et al., 2006). The addition of DNase to growth medium inhibits biofilm formation at early stages, suggesting that DNA is important for biofilm development (Whitchurch et al., 2002). However, the addition of DNase to established biofilms does not significantly disrupt them due, at least partly, to protective interactions within the biofilm matrix.

## Use of Clsm to Identify Contributions of *P. Aeruginosa* Matrix Components

A study by Colvin et al. investigated the matrix composition of a panel of environmental and clinical *P. aeruginosa* isolates.Within this study, we identified the roles that the EPS Psl and Pel play in biofilm formation (Colvin et al., 2012). Two different biofilm culturing methods were used. In the first method, bacteria were cultured statically in microtiter plates. The amount of adherent biofilm biomass is determined by staining with crystal violet dye and quantifying the absorbance of bound dye, following extensive washing (O’Toole and Kolter, 1998). This method is useful since it is relatively high-throughput. However, fragile biofilms are susceptible to disruption during the wash steps, and furthermore, the assay does not provide any information about biofilm structure. The second approach allows for visualization of the biofilm aggregate structures using CLSM. In this assay, biofilms are cultured in dilute medium under continuous flow in a flow-cell reactor, and live biofilms are imaged.

Initially, a panel of isolates and their isogenic single and double Δ*psl* and Δ*pel* mutants were tested for surface attachment and static biofilm formation. We discovered that Psl was important for surface attachment in most strains, but there was strain-to-strain variation in the contribution of Psl and Pel to biofilm aggregate formation. For the common laboratory strain PA14, which only produces Pel as its EPS, as expected Pel was necessary for biofilm formation. Similarly, for another common laboratory strain PAO1, Psl was necessary for biofilm formation and Pel was dispensable. Five additional isolates similarly required Psl and not Pel for biofilm formation. The five additional isolates that were analyzed could use either Psl or Pel to form biofilms, and deleting only *psl* or *pel* did not diminish static biofilm formation relative to the parental isolate. Finally, we observed isolates that exhibit overproduction of both Psl and Pel. Two of these isolates produced copious amounts of biofilm relative to the other isolates. Based upon these results, we categorized strains as: (1) Pel-dominant matrix, (2) Psl-dominant matrix, (3) EPS-redundant users, and (4) matrix over-producers.

Representatives from these classes were then analyzed further using microscopy. In general, these results matched the results of the static biofilm assay. However, important structural information was gleaned. As had been shown previously, both of the common laboratory strains, PA14 and PAO1, formed large aggregates that adhered to the flow-cell cover slip. This aggregation was dependent upon expression of their primary EPS, Pel for PA14 and Psl for PAO1. One “Psl-dominant” isolate, E2, formed thick, rough biofilms without distinct aggregates. As expected based upon the static biofilm assay results, the Δ*pel* mutant formed similar biofilms to the parental strain, but both the Δ*psl* and Δ*psl* Δ*pel* mutants were severely attenuated for biofilm formation, and only a few cells adhered. In contrast, another “Psl-dominant” isolate, S54485, displayed different characteristics. This strain and its Δ*pel* mutant similarly formed thick biofilms without distinct aggregates. However, although the amount of adherent biomass for the Δ*psl* mutant was much less than the parental strain, it formed small aggregates, suggesting that Pel may play more of role in the biofilm formation of this strain than was recognized based upon the static biofilm results alone. In this way, microscopy was a useful tool to understand differing biofilm matrix requirements of different isolates.

The EPS-redundant and matrix-overproducing strains that were analyzed by CLSM of flow-cell biofilms also displayed interesting phenotypes that were not evident from the static biofilm assay. For example, one EPS-redundant isolate MSH3 forms large aggregates that appear similar to PAO1. Based upon the static-biofilm assay, we did not anticipate that either Δ*psl* or Δ*pel* mutations would change its biofilm formation. However, the Δ*pel* mutant formed smaller aggregates, and the Δ*psl* mutant did not form any aggregates and appeared similar to the Δ*psl* Δ*pel* mutant. This result suggested that while both Psl and Pel may help this isolate to form adherent biomass, Psl appears to be more necessary to assemble structured aggregates. One of the matrix-overproducing isolates studied, 19660, also displayed interesting structural characteristics. Both the parental strain and the Δ*pel* mutant formed many aggregates on the surface of the flow-cell cover slip. However, aggregates formed by 19660 Δ*psl* had sparser coverage but were much larger. This study provided information about the ability of isolates to use either Psl or Pel to form adherent biomass, and also highlighted that each of these EPS can play distinct roles in structuring biofilm aggregates. These results also illustrate that knowing the predominant matrix EPS is not a good predictor of biofilm structure. As described in the following sections of this review, CLSM of flow-cell biofilms has been used to provide mechanistic understanding of how the matrix interactions and biofilm localizations of Psl and Pel result in their overlapping and unique biofilm roles.

## Localization of Matrix Components Using Fluorescence Microscopy

In an earlier study by Ma et al., Psl localization was visualized throughout PAO1 biofilm development (Ma et al., 2009). For these experiments, biofilms were cultured under continuous flow in flow-cell biofilm reactors and then imaged by CLSM. Psl was stained using specific fluorescently conjugated lectins: *Marasmium oreades* agglutinin (MOA) or (*Hippeastrum hybrid* agglutinin (HHA). These lectins were shown to be specific for Psl in a previous study (Ma et al., 2007). The bacterial cells were either counterstained with the membrane stain FM4-64 or visualized *via* expression of the fluorescent protein GFP.

Psl localization changed during the course of biofilm development. At early timepoints, Psl was observed associated with surface-attached bacterial cells. Psl remained associated with early aggregates, and is thought to be important for cell-cell interactions and matrix assembly. As the biofilm matured, larger aggregates formed. Psl accumulated at the periphery and was not observed in the interior cavity of these aggregates. The peripheral localization of Psl was readily observed in optical cross-sections of z-stacked images of the bacterial aggregates. Several experiments were performed to verify that the peripheral Psl staining was not due to inability of the Psl-specific lectin to access the aggregate interior. First, the concentration of lectin was increased, and the staining incubation time was also increased, neither resulted in staining of the aggregate interior. Next, to verify that the lectin was not excluded based on its size, FITC-conjugated dextran beads (slightly larger than the lectin) were introduced into the aggregate, and these were able to access the interior of the biofilm aggregates. Thus, the peripheral staining of Psl was indeed due to localization of Psl and not due to an experimental artifact.

Past studies showed that *P. aeruginosa* biofilm matrix contained high levels of eDNA (Whitchurch et al., 2002). To determine if Psl and eDNA co-localized in the aggregate, mature aggregates were stained with both HHA-FITC and the nucleic acid stain propidium iodide. Interestingly, the interior cavity of the aggregates stained with the propidium iodide. Thus, Psl and eDNA occupy distinct regions of biofilm aggregates. Additionally, Psl was observed at all stages although its localization varied with biofilm development. The mechanism of Psl localization is still under study.

## Interactions Between Matrix Components

The functional impact of interactions between different matrix components can be observed using CLSM. This approach helped us to understand matrix interactions that occur with the EPS in non-mucoid *P. aeruginosa* biofilms. In the first study described below, the functional impact of Psl-binding to CdrA was discovered. In the second case, the functional impact of the cationic EPS Pel was discovered, including that Pel binds to eDNA in biofilm aggregates.

### CdrA Is a Matrix Protein That Promotes Aggregation and Helps to Retain the EPS Psl

Borlee et al. identified CdrA as a key matrix protein and used a suite of approaches to elucidate its biofilm role (Borlee et al., 2010). CdrA initially was identified as a likely biofilm protein in a screen of factors that were expressed by *P. aeruginosa* under conditions of high levels of the intracellular second messenger cyclic di-GMP (c-di-GMP) (Starkey et al., 2009). Other genes important for biofilm formation, including for the EPS Psl and Pel, are also upregulated under conditions of high c-di-GMP. This study verified that *cdrA* transcription was c-di-GMP dependent, and also found that *cdrA* is part of a two gene operon, *cdrAB*. This operon encodes a two-partner secretion system (TPSS) in which CdrA is the cargo and CdrB is the outer-membrane pore required for export of CdrA from the periplasm to cell surface. CdrA is encoded as a 220 kDa protein. Two forms of CdrA were observed by Western blot analysis: a 220-kDa form in the cellular fraction and a 150-kDa processed form in the culture supernatant fraction. More recent studies showed that the periplasmic protease LapG is responsible for cleaving CdrA from the cell-surface, resulting in the release of CdrA into the culture supernatant (Rybtke et al., 2015; Cooley et al., 2016).

Based upon its regulation by c-di-GMP, it was hypothesized that CdrA may contribute to the *P. aeruginosa* biofilm matrix. First, the contribution of CdrA to static biofilms was assayed using a crystal violet assay. A Δ*cdrA* mutation was found to have minimal impact on the amount of static biofilm formed in either the wild-type PAO1 or PAO1 Δ*wspF* background. However, EPS can mask the effects of bacterial adhesins, and so the assay was also performed for PAO1 Δ*wspF* Δ*psl* Δ*pel*. In this case, the Δ*cdrA* mutation had much less adherent biomass. The overexpression of *cdrAB* was also tested, and we observed a large (6.4-fold) increase in static biofilm formation. Together, these results suggested that CdrA could promote biofilm formation.

While CdrA-deficient biofilms still accumulated biomass as determined by the crystal violet assay, a much stronger CdrA-dependent phenotype was observed for biofilms cultured under continuous flow. For this experiment, *P. aeruginosa* biofilms were cultured in flow-cells and then the resulting biomass was stained with Syto9 and imaged by CLSM. It was observed that relative to the wild-type PAO1 strain, PAO1 Δ*cdrA* formed biofilm aggregates that were shorter in height and smaller in diameter. The loss of CdrA in the biofilm matrix-overproducing strain PAO1 Δ*wspF*, which typically forms large aggregates and high levels of overall biomass, was also tested. As viewed by CLSM, PAO1 Δ*wspF* Δ*cdrA* formed only loosely assembled aggregates, which were easily dislodged from the flow-cell surface by altering the flow rate.

There were some clues as to the mechanisms by which CdrA promoted biofilm assembly and structurally reinforced the biofilm aggregates. Based upon computational modeling, CdrA was predicted to be structurally similar to other TPSS proteins such as filamentous hemagglutinin (FHA), including a β-helical motif that makes up the elongated fibrillar protein core. Like FHA, the CdrA structure is predicated to contain sugar binding and carbohydrate-dependent hemagglutination domains, and it was thought that these domains may be important for its interactions with EPS. This led us to predict that CdrA may promote biofilm formation by tethering Psl to the bacteria or by crosslinking of CdrA and Psl. Several experiments were performed to test if CdrA interacted with EPS. First, it was observed that overexpression of *cdrAB* resulted in aggregation in liquid culture. This aggregation was most pronounced when the EPS Psl was also made, and the aggregation could be reduced approximately 60% by the addition of mannose (a Psl constituent monosaccharide) to the culture medium. As additional supportive evidence of interactions between CdrA and Psl, it was found that CdrA co-immunoprecipitated (Co-IP) with Psl from liquid culture supernatant. Finally, CLSM was used to monitor the impact of CdrA on Psl localization in biofilms grown under continuous flow. As was observed previously for PAO1 Δ*wspF*, Psl was tightly associated with the periphery of biofilm aggregates. Interestingly, this localized retention of Psl was CdrA-dependent, and aggregates formed by the PAO1 Δ*wspF* Δ*cdrA* strain did not have tightly associated Psl. Thus, CdrA promotes biofilm formation, and does so, at least in part, through its retention of Psl.

### Interactions Between the EPS Pel and eDNA

Recently, Jennings et al. extended this microscopy method to characterize the *P. aeruginosa* EPS Pel (Jennings et al., 2015). Until recently, Pel was mistakenly believed to be a glucose-rich molecule. This misidentification was due in part to the recalcitrance of Pel to isolation and standard preparatory hydrolysis conditions for monosaccharide compositional analysis. Using harsher hydrolysis conditions for monosaccharide compositional analysis, Pel was determined to be a polymer of N-acetylglucosamine (GlcNAc) and N-acetylgalactosamine (GalNAc). This assignment was supported by the reactivity of Pel to antibodies raised against poly-β-1,6-N-acetylglucosamine (PNAG) and chitosan (poly-β-1,4-N-acetylglucosamine) as well as binding to the GalNAc-specific lectin *Wisteria floribunda* lectin (WFL). Additionally, using anion exchange chromatography, Pel was found to be positively charged, which was hypothesized to be due to partial deacetylation.

Determination of Pel composition allowed for the design of microscopy methods to further characterize Pel functionality. Specifically, the combination of CLSM and staining of Pel with fluorescently conjugated WFL permitted the direct visualization of Pel localization in biofilm aggregates. Interestingly, Pel localization was found to be strain specific. In the Psl-dominant strains PAO1 and PAO1Δ*wspF*, minimal Pel staining was observed. However, some Pel staining was observed in the stalks of the aggregates. In contrast, in the four Pel-dominant strains studied (PA14, PA14Δ*wspF*, PAO1Δ*wspF* Δ*psl*, and PAO1Δ*wspF* Δ*psl* pBAD*pel*), Pel localized to both aggregate periphery and stalk. Importantly, staining with WFL was not observed in Δ*pel* strains. As mentioned earlier, it previously was shown that Psl localizes to the aggregate periphery. While Psl is the dominant EPS at the periphery, in its absence, Pel is able to compensate. Taken together with the Pel findings, this suggested that aggregates may require EPS at their periphery. To describe the idea that a growing aggregate would have to continuously remodel the peripheral EPS shell to accommodate new biomass, we have coined as the “expanding balloon hypothesis.”

The unique localization of Pel to the aggregate stalk was explored. This region had previously been shown to be rich in eDNA. Interestingly, crude preparations of Pel were often contaminated with eDNA. Together, these results suggested that Pel and eDNA may interact to provide structure to *P. aeruginosa* biofilms. To test Pel-DNA binding, Pel-containing supernatants were mixed with exogenous salmon sperm DNA and then monitored for aggregation. Indeed, visible aggregates formed that could be stained with the dye Congo red, which is known to bind to Pel. Large aggregates did not form if Δ*pel* supernatants were used or in the absence of exogenous DNA. Furthermore, aggregation was pH-dependent; aggregation was only observed if the assay was performed at a pH at or below the isoelectric point of Pel (pH 6.3) when Pel would be positively charged. This result supported the prediction that Pel and eDNA interact due to the cationic nature of Pel. To determine if Pel and eDNA interacted in biofilm aggregates, two microscopy experiments were performed. In the first experiment, localizations of Pel and endogenous eDNA were monitored by again staining Pel with fluorescently conjugated WFL and staining eDNA with propidium iodide. Imaging of multiple matrix components in the same experiment requires the use of spectrally resolved fluorescent probes. Initial trials were performed to stain for eDNA with propidium iodide (red fluorescence) and Pel with fluorescein-conjugated WFL (green fluorescence). However, it was not possible to stain the same biofilm for both Pel and eDNA, possibly due to interactions between the two stains. While it may have been possible to optimize the choice of lectin and/or nucleic acid stain, in this study, Pel and eDNA were stained in separate biofilms, and it was discovered that both Pel and eDNA co-localized to the biofilm aggregate stalk. In a separate experiment, exogenous salmon sperm DNA was added to established biofilms formed by PAO1Δ*wspF* Δ*psl* pBAD*pel* (overexpresses Pel) and the negative control PAO1Δ*wspF* Δ*pel* pBAD*psl* (overexpresses Psl). DNA accumulation was monitored by staining with propidium iodide. The exogenous DNA accumulated in the stalk of the biofilm aggregates formed by PAO1Δ*wspF* Δ*psl* pBAD*pel*, and did not accumulate in the negative control biofilms, suggesting that the accumulation was Pel specific. The DNA did not bind to peripherally located Pel, and it was suggested that this may be due to differences in pH (and thus Pel acetylation state) between the interior and exterior environments of the aggregate.

In summary, this study identified the composition of a key *P. aeruginosa* EPS Pel, which permitted the biofilm functionality of Pel to be determined. It was found that Pel is a positively charged polysaccharide, and this characteristic influences its co-localization with eDNA at the stalk of the biofilm aggregate.

## Use of Microscopy to Determine Matrix Functionality

We have also used CLSM to map spatiotemporal impacts of antimicrobial treatment and the protective functionality of the biofilm matrix. Biofilm cells are less susceptible to antibiotics compared to planktonic bacteria (Stewart and Costerton, 2001). There are multiple reasons for this phenomenon. Although not believed to be the primary reason for decreased susceptibly, in some cases, the biofilm matrix can impede diffusion of antimicrobials, preventing the drugs from reaching their intended target at the surface of or within the bacteria. In some instances, biomineralization that can occur in biofilms modulates diffusion of molecules such as antimicrobials into the biofilm aggregate (Li et al., 2016; Keren-Paz et al., 2018). It is appreciated that biomineralization occurs in biofilms formed by *P. aeruginosa*, *Bacillus subtilis,* and many other bacteria (Li et al., 2015, 2016; Oppenheimer-Shaanan et al., 2016; Keren-Paz et al., 2018). The deposition and structure of minerals, such as the crystalline calcium carbonate (calcite) that occurs in *P. aeruginosa* biofilms, has been examined using a range of techniques including Raman spectroscopy and micro-CT X-ray imaging, and calcein staining was used to visualize calcite with CLSM in *P. aeruginosa* biofilms formed in flow-cells (Li et al., 2016; Keren-Paz et al., 2018). Past studies determining drug penetration into biofilms were mostly performed using mucoid *P. aeruginosa* biofilms (biofilms that produce alginate as their primary EPS) (Gordon et al., 1988). In the study described below, we used CLSM to determine that some antibiotics are only able to access and kill bacteria positioned at the surface of biofilm aggregates. This finding was important because it highlighted a mechanism through which biofilm bacteria are protected from antibiotics.

### The Biofilm Matrix Protects Against Antibiotic Treatment

In this study by Tseng et al., CLSM of flow-cell biofilms was used to investigate if antibiotics were able to penetrate non-mucoid *P. aeruginosa* biofilms (Tseng et al., 2013). For this study, two clinically important antibiotics, the aminoglycoside tobramycin and the fluoroquinolone ciprofloxacin, were studied. PAO1 biofilms were cultured in flow-cell biofilm reactors and imaged after formation of large aggregates that were several micrometers high. PAO1 bacteria that constitutively expressed GFP were used to allow for the bacteria to be fluorescently imaged. The biofilms were treated with Cy5-labeled antibiotics and the Cy5-only control. Penetration of the antibiotics was visualized as Cy5 intensity over the course of 30 min of static incubation followed by 30 min of washing. Both Cy5-alone and Cy5-ciprofloxacin rapidly penetrated the biofilm aggregates during the static incubation and then were easily removed during the wash period. This result suggested that neither interacted strongly with the biofilm. In contrast, Cy5-tobramycin accumulated at the periphery of the aggregate, and it was not removed by washing. This suggested that tobramycin interacted with a biofilm component present at the aggregate periphery.

As mentioned earlier, depending upon the bacterial strain, both Psl and Pel can localize specifically to the biofilm aggregate periphery (Ma et al., 2009; Jennings et al., 2015). To test if tobramycin was retained at the periphery by one of the EPS, we monitored penetration of Cy5-tobramycin into aggregates formed by PA14 (unable to produce Psl), PAO1 Δ*pelF* (unable to produce Pel), and PAO1 Δ*algD* (unable to produce alginate). Cy5-tobramycin was similarly retained at the periphery of aggregates formed by all of these strains, suggesting that tobramycin was not interacting with EPS. Other non-matrix possibilities were explored, but an interacting molecule was not identified. To determine if an interaction between some biofilm component and tobramycin was responsible for the localized retention of the antibiotic, two experiments were performed. In the first experiment, biofilms were treated with Cy5-tobramycin and increasing concentrations of unlabeled tobramycin. Incubation with high concentrations of unlabeled tobramycin resulted in an increase in the penetration of Cy5-tobramycin into the aggregate. This result suggested that the tobramycin binding sites could be saturated. In the second experiment, the nature of the interaction was explored. Because tobramycin is positively charged, we predicted that retention of tobramycin was due to an ionic interaction between the drug and biofilm. To test this, penetration of Cy5-tobramycin was monitored in the presence of increasing concentrations of a metal cation (Mn^2+^). Indeed, incubation with the cation permitted increased penetration of Cy5-tobramycin into the aggregate, supporting our hypothesis that the positively charged tobramycin was retained at the biofilm aggregate periphery by a negatively charged matrix component.

To determine if the localization of tobramycin impacted bacterial death in the aggregate, a tobramycin response reporter was constructed. For this reporter, expression of chromosomally integrated *gfp* was driven by the promoter of *ibpA*, which encodes a protein that is induced upon treatment with tobramycin (Kindrachuk et al., 2011). The reporter showed activity (measured as fluorescence intensity) after exposure to tobramycin, and the activity increased with increasing tobramycin concentrations. The reporter activity was limited to the peripheral region, where tobramycin was bound. This suggested that non-mucoid *P. aeruginosa* biofilms can prevent the penetration of some antibiotics, which protects some of the biofilm bacteria from exposure to the drug.

### The Biofilm Matrix-Associated Protease Inhibitor Ecotin Protects *P. aeruginosa* From Attack by Neutrophil Elastase

Proteins associated with the biofilm matrix of bacterial aggregates have been thought to provide many important functions to the community. To identify matrix proteins, a protocol was developed, which was based on a protocol used by Absalon and colleagues in *Vibrio cholerae* (Absalon et al., 2011). Briefly, the extracellular proteins of mature PAO1 biofilms were biotinylated prior to biofilm disruption, and then the biotinylated proteins were purified and identified using mass spectrometry (Tseng et al., 2018).

The study by Tseng et al., characterized one identified matrix protein called ecotin (Tseng et al., 2018). Ecotin is able to inhibit neutrophil elastase, a bactericidal enzyme produced by the host immune system during *P. aeruginosa* infections. *Via* immunoblotting, it was found that while ecotin was present in the whole biofilm at all timepoints, ecotin was only found at high levels in the extracellular matrix in older biofilms. This increase in ecotin in the matrix was associated with a corresponding increase in the amounts of the matrix polysaccharide Psl. A Co-IP assay was used to determine that ecotin binds to Psl. Furthermore, it was determined that Psl-bound ecotin was enzymatically active and able to inhibit neutrophil elastase activity.

We predicted that matrix-bound ecotin would protect *P. aeruginosa* from neutrophil elastase-mediated killing. To test this hypothesis, static glass-grown biofilms were treated with neutrophil elastase and the percentage of bacteria killed by the treatment was determined using viability staining and imaging by CLSM. It was found that Δ*eco* biofilms were more susceptible to killing than wild-type biofilms. Also, complementation of *eco* rescued the biofilm susceptibility defect. To isolate the contribution of matrix-bound ecotin, it was examined whether exogenous addition of purified ecotin could protect Δ*eco* biofilms from neutrophil elastase-mediated killing. For this experiment, purified ecotin was applied to static glass-grown biofilms, which then were rinsed to remove any ecotin that did not bind to the matrix. Biofilms that were treated with purified ecotin were significantly protected from neutrophil elastase treatment. The protective effect of exogenous ecotin was not observed if the bacteria did not make Psl, which is believed to be because only Psl-positive biofilm matrix appreciably binds to ecotin. This study used microscopy to identify the protective function of a matrix protein ecotin, which binds to Psl.

## Conclusions and Future Perspectives

Understanding of the structural changes that biofilms undergo throughout their lifecycle requires an understanding of the structure and function of the individual biofilm matrix components as well as of how those biomolecules assemble into a three-dimensional architecture. We have used CLSM to investigate that biofilm matrix biomolecules have structural and chemical properties that are specific to their function. For example, matrix components may possess properties that mediate interactions with other biomolecules (i.e., Pel and eDNA interactions) to produce unique properties that support structure and function of the entire biofilm (Borlee et al., 2010; Jennings et al., 2015). As such, investigations which use CLSM to complement additional microbiological approaches have identified the structural and functional basis of biofilm matrix stability and function. This improved understanding will provide more nuanced approaches to treating biofilm infections such as strategies to disrupt biofilms so that biofilm bacteria become susceptible to antimicrobial treatment and host immune responses.

There are many questions that remain unanswered about the *P. aeruginosa* biofilm matrix. For example, as of yet, the localization of matrix proteins in *P. aeruginosa* has not been determined. One could imagine that the correct spatial positioning of a matrix protein might be crucial for reaping the benefits of its extracellular activity. Unfortunately, we currently lack the resolution to resolve the positioning of matrix proteins relative to other components. The use of fluorescently conjugated antibodies to stain proteins in biofilms could be used to determine if Psl-binding proteins (such as CdrA and ecotin) co-localize with Psl during biofilm development. Additionally, advances in super resolution microscopy have permitted the tracking of single matrix components for *Vibrio cholerae* (Berk et al., 2012), but similar methods have not been applied to *P. aeruginosa*. Another method that may be useful is electron microscopy, which has been applied to investigate overall biofilm and biofilm matrix morphology (Hung et al., 2013; Serra et al., 2013; Hollenbeck et al., 2014; Joubert et al., 2017). For electron microscopy, immunogold-labeled antibodies can be used to study matrix proteins.

Indeed, super resolution fluorescence microscopy might lead to novel insight concerning matrix structure. The one study to date that has been performed led to the discovery that *V. cholerae* precisely positions its matrix EPS and proteins (Berk et al., 2012). Investigating the functional significance of such positioning, including mechanisms through which it is localized, is an obvious next step in understanding biofilm assembly. For *P. aeruginosa*, another interesting vista is understanding the advantages and constraints of using different EPS components. We might very well find that *P. aeruginosa* finely tunes EPS production in response to the environment as a means to maximize the functionality of the matrix.

## Author Contributions

CR wrote the first draft of the manuscript. CR and MP contributed to manuscript revision, read, and approved the submitted version.

## Conflict of Interest Statement

The authors declare that the research was conducted in the absence of any commercial or financial relationships that could be construed as a potential conflict of interest.
